# Exploratory Evaluation of a Sodium Iodide-Based Root Canal Filling Formulation in a Canine Model of *Enterococcus faecalis*-Induced Periapical Inflammation

**DOI:** 10.3390/pharmaceutics18040493

**Published:** 2026-04-17

**Authors:** Saeromi Jun, Sak Lee, Jong-Soo Kim, Min-Cheol Song, Ji-Sun Shin, Yu-Jin Kim, Jung-Wook Kim, Jung-Hwan Lee

**Affiliations:** 1 Program in Pediatric Dentistry, Department of Dental Science, Graduate School, Seoul National University, 101 Daehak-ro, Jongno-gu, Seoul 03080, Republic of Korea; saemi21@naver.com; 2 Department of Pediatric Dentistry, Dankook University Jukjeon Dental Hospital, 152 Jukjeon-ro, Yongin 16890, Republic of Korea; 3 Department of Oral Pathology, Seoul National University Dental Hospital, Seoul 03080, Republic of Korea; leesak@snu.ac.kr; 4 Department of Oral Pathology, School of Dentistry, Seoul National University, Seoul 03080, Republic of Korea; 5 Department of Pediatric Dentistry, College of Dentistry, Dankook University, 119 Dandae-ro, Cheonan 31116, Republic of Korea; jskim@dku.edu (J.-S.K.); pedoshin@dankook.ac.kr (J.-S.S.); 6 Department of Conservative Dentistry, Dankook University Jukjeon Dental Hospital, 152 Jukjeon-ro, Yongin 16890, Republic of Korea; smch_izzim3@naver.com; 7 Institute of Tissue Regeneration Engineering (ITREN), Dankook University, 119 Dandae-ro, Cheonan 31116, Republic of Korea; yujin10316426@gmail.com; 8 Department of Biomaterials Science, College of Dentistry, Dankook University, 119 Dandae-ro, Cheonan 31116, Republic of Korea; 9 Department of Pediatric Dentistry, Dental Research Institute, School of Dentistry, Seoul National University, 101 Daehak-ro, Jongno-gu, Seoul 03080, Republic of Korea; 10 Department of Molecular Genetics, Dental Research Institute, School of Dentistry, Seoul National University, 101 Daehak-ro, Jongno-gu, Seoul 03080, Republic of Korea; 11 Department of Nanobiomedical Science, BK21 PLUS NBM Global Research Center for Regenerative Medicine, Dankook University, 119 Dandae-ro, Cheonan 31116, Republic of Korea; 12 UCL Eastman-Korea Dental Medicine Innovation Centre, Dankook University, 119 Dandae-ro, Cheonan 31116, Republic of Korea; 13 Cell & Matter Institute, Dankook University, 119 Dandae-ro, Cheonan 31116, Republic of Korea; 14 Mechanobiology Dental Medicine Research Center, Dankook University, 119 Dandae-ro, Cheonan 31116, Republic of Korea

**Keywords:** sodium iodide, root canal filling material, dog model, *Enterococcus faecalis*, periapical inflammation, Vitapex, Calcipex

## Abstract

**Background and Objectives**: Premature loss of primary teeth can disrupt occlusal development and oral function. Although iodoform-based materials such as Vitapex^®^ are widely used, concerns remain regarding their cytotoxicity and potential to accelerate root resorption. Sodium iodide (NaI) has emerged as a biocompatible, antibacterial alternative. This study evaluated the feasibility of a NaI-based root canal filling material in a canine model of *Enterococcus faecalis*-induced periapical inflammation. **Methods**: Periapical lesions were induced in a healthy male mongrel dog using *E. faecalis* (10^6^ CFU/mL). After six weeks, the root canals were obturated with NaI paste, Vitapex^®^, or Calcipex. Untreated teeth and an *E. faecalis*-only group served as controls. Radiographic lesion sizes were monitored at 4, 8, 12, and 16 weeks post-obturation. Histological analysis at 16 weeks assessed inflammatory area and perimeter, stromal fibrosis, inflammatory cell infiltration, and myeloperoxidase (MPO) expression. **Results**: Radiographically, all treatment groups showed reduced lesion size relative to the positive control. No significant differences were observed among the NaI, Vitapex^®^, and Calcipex groups at 4 and 8 weeks; however, significant differences emerged at 12 and 16 weeks (*p* < 0.05). The NaI group showed lesion reduction until week 8, followed by subsequent expansion thereafter, whereas the Vitapex^®^ and Calcipex groups showed continuous lesion reduction over time. Histologically, the periapical inflammatory area increased in the order of Vitapex^®^ < Calcipex < NaI < positive control (*p* < 0.05). MPO staining identified neutrophils as the primary inflammatory cells. **Conclusions**: NaI paste showed favorable early radiographic healing but limited long-term stability compared with conventional materials. With further optimization, it may have potential as an alternative root canal filling material. However, given the single-animal exploratory design, these findings should be interpreted as preliminary rather than definitive evidence.

## 1. Introduction

Premature loss of primary teeth can negatively affect the overall oral health of children, including occlusal development, maintenance of space for permanent tooth eruption, speech, and mastication. Therefore, root canal treatment that preserves the longevity of primary teeth while remaining compatible with physiological root resorption represents an important clinical challenge in pediatric dentistry [1]. In particular, the choice of root canal filling material plays a critical role in treatment outcomes, as its biocompatibility and root resorption pattern directly affect long-term prognosis [2].

Iodoform-containing calcium hydroxide pastes, including Vitapex^®^ and Metapex^®^, are among the commonly used root canal filling materials for pulpectomy in primary teeth [3]. Clinicians have long used these materials because of their advantages, including ease of use, antibacterial properties, and radiopacity. However, iodoform-based medicaments have been reported to negatively affect tissue healing and induce fibrous tissue formation [4]. More recently, concerns have been raised that these components may cause tissue toxicity and accelerate root resorption in primary teeth, leading to premature exfoliation [5,6,7]. Such outcomes may adversely affect the normal timing of permanent tooth eruption and occlusal development. Consequently, there is an increasing need to develop novel root canal filling materials as alternatives to iodoform.

Within this context, sodium iodide (NaI) has been proposed as a potential substitute. As an iodine-based compound, NaI retains antibacterial properties [8,9] but may elicit biological responses different from those of conventional iodoform-based materials. Recent studies have suggested that NaI-containing formulations exhibit a relatively lower capacity to promote osteoclast differentiation, which may help prevent accelerated root resorption [10]. Furthermore, studies have shown that its cytotoxicity remains within acceptable limits [11], indicating its potential to prolong the retention period of primary teeth.

In addition, evaluations of the physicochemical properties of NaI-based filling materials have demonstrated improvements in solubility and ion release characteristics compared with existing formulations [12]. Several studies have also attempted to enhance formulation stability through material modifications. Preclinical animal studies (dog tooth models) have shown relatively favorable histological responses in periapical tissues when using NaI-based filling materials, with outcomes comparable to or better than those of iodoform-based compositions [13]. These findings support the potential of NaI-based root canal filling materials as a viable clinical alternative.

Nevertheless, existing studies have largely been confined to basic in vitro experiments or limited preclinical models. Evidence regarding long-term tissue responses and clinical stability under bacterially induced periapical lesion conditions remains insufficient. In particular, further investigations are required to determine how root canal filling materials influence immune and inflammatory responses under infected conditions, as well as their effects on root resorption and periapical healing processes observed in clinical practice.

*Enterococcus faecalis* (*E. faecalis*) is a key pathogen contributing to root canal treatment (RCT) failure. It demonstrates resistance to calcium hydroxide [14], forms robust biofilms on root canal walls that hinder disinfectant penetration [15], and may thereby cause reinfection following treatment [16,17].

Therefore, this preliminary preclinical study aimed to assess the in vivo feasibility of a sodium iodide (NaI)-based root canal filling material in a canine model of *E. faecalis*-induced periapical inflammation and to compare its radiographic and histological outcomes with those of conventional materials.

## 2. Materials and Methods

### 2.1. Animal Study

#### 2.1.1. Ethical Approval and Compliance

This animal experiment was reviewed and approved by the Institutional Animal Care and Use Committee (CRONEX-IACUC), project CRONEX-IACUC:202410004, on 26 October 2024. Informed consent from owners was not required as no privately owned animals were involved.

#### 2.1.2. Reporting Guidelines

The procedures were designed and reported in accordance with the PRIASE 2021 guidelines [18] for reporting animal studies in Endodontology and the ARRIVE 2.0 guidelines [19], and were conducted under the ethical standards of ISO 7405:2018 [20] for the biological evaluation of dental materials. In accordance with the PRIASE 2021 guidelines, a flowchart summarizing the experimental design and animal procedures is presented in Supplementary Figure S1, and the detailed PRIASE 2021 checklist is provided as Supplementary Table S1. The ARRIVE 2.0 Author Checklist is provided as Supplementary Table S2.

#### 2.1.3. Environmental Conditions and Postoperative Care

The animal’s health status was regularly monitored by a licensed veterinarian. The animal was maintained in a controlled environment at 22 ± 2 °C with 50 ± 10% relative humidity and under 100% HEPA-filtered air ventilation. A 12-h light–dark cycle was used (lights on 08:00–20:00) with an ambient illuminance of approximately 200 lux. Background noise levels were ≤45 dB, and ammonia concentrations were ≤20 ppm throughout the housing area.

After each operative session, the animal received Convenia^®^ (Zoetis Inc., Kalamazoo, MI, USA) at 8 mg/kg (0.1 mL/kg) and Metacam^®^ (Boehringer Ingelheim Animal Health USA Inc., Duluth, GA, USA) at 0.1 mL/kg according to the institutional analgesia/antibiotic protocol.

#### 2.1.4. Sample Size and Allocation

**Figure 1:**
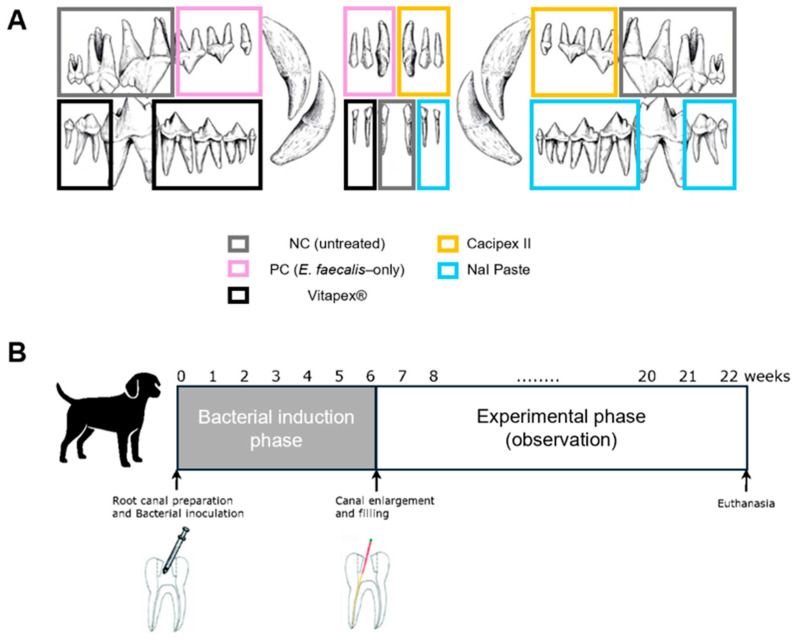
Summary of a dog model study evaluating the effect of a novel NaI-based root canal filling material on periapical inflammation. (**A**) Classification of the experimental groups. Thirty-six healthy teeth with fully developed roots were selected (18 maxillary and 18 mandibular teeth), including incisors, premolars, and molars. (**B**) Experimental procedure. The study was conducted over a total of 22 weeks, including 6 weeks for the induction of periapical inflammation through bacterial infection and 16 weeks for the observation of changes in lesion size after root canal filling.

A clinically healthy male mongrel dog, 12 months of age and weighing approximately 40 kg, was selected as the experimental subject. Clinical and radiographic examinations were performed to identify healthy teeth with fully developed roots and no signs of periapical lesions or abnormal mobility. Teeth that were morphologically unsuitable for endodontic treatment or difficult to evaluate radiographically were excluded from the study. Based on these evaluations, a total of 36 teeth were included in the study; nine teeth were selected from each quadrant (upper right, upper left, lower right, and lower left). These included anterior, premolar, and molar teeth (Figure 1). Because of the limited number of experimental units, randomization was not performed. Instead, teeth were allocated with consideration of tooth characteristics, including root configuration (single-rooted vs. multi-rooted) and tooth category, so that the anterior teeth, premolars, and molars were represented across the experimental groups. In addition, all procedures were performed by the same experienced pediatric dentist according to a standardized protocol to reduce operator-related variability.

### 2.2. Materials

#### 2.2.1. Induction of Periapical Inflammation

To induce periapical inflammation, *Enterococcus faecalis* (*E. faecalis*, ATCC 19433) was purchased from the American Type Culture Collection (ATCC; Manassas, VA, USA). The strain, preserved in glycerol stock at −80 °C, was streaked onto brain heart infusion agar (BHIA; BD Difco, Franklin Lakes, NJ, USA) plates and incubated at 35 ± 2 °C for 18 h. After incubation, a single colony was transferred to brain heart infusion broth (BHIB; BD Difco, Franklin Lakes, NJ, USA) and cultured with shaking at 35 ± 2 °C for 18 h. The resulting bacterial suspension was then diluted with BHIB to obtain a cell density of approximately 1 × 10^6^ CFU/mL [11]. A 100 µL aliquot of a 10^6^ CFU/mL suspension was inoculated into each prepared root canal, following the infection protocol described in Section 2.3.

#### 2.2.2. Experimental Materials and Group Allocation

A novel root canal filling material composed of calcium hydroxide, sodium iodide, silicone oil, and lanolin was used as the experimental material. The material was prepared under aseptic conditions by hand-mixing the individually sterilized components on a sterile glass slab with a sterile spatula inside a clean bench. This formulation has previously been shown to meet the physicochemical criteria established by the International Organization for Standardization (ISO) in accordance with ISO 6876:2012 [21]. For comparison, two commercially available root canal filling materials were included: Calcipex II and Vitapex^®^. The composition of each material is presented in Table 1. Based on the type of material applied in each canal, the teeth were divided into five experimental groups (Figure 1).

### 2.3. Methods

#### 2.3.1. Animal Preparation and Anesthesia

The dog was fasted overnight prior to the procedure. General anesthesia was induced via intramuscular injection of Zoletil^®^ 50 (Virbac, Carros, France) at a dose of 4 mg/kg combined with Rompun^®^ (Bayer, Leverkusen, Germany) at 2.2 mg/kg. Following induction, the animal was transported to the laboratory, positioned on an operating table, and connected to a veterinary anesthesia machine (Fabius^®^ GS Premium, Dräger, Germany). Inhalation anesthesia was maintained with sevoflurane (Vitapure, Mumbai, India) and oxygen. Vital signs including heart rate and oxygen saturation were continuously monitored using a patient monitoring system (Vista 120, Dräger, Germany) to ensure anesthetic stability.

To ensure stable access, the jaws were maintained in an open position by securing the maxilla and mandible to a vertical support stand using elastic compression bandages. Full-mouth clinical photographs and periapical radiographs were taken. The oral cavity was irrigated with hydrogen peroxide and sterile saline, followed by local anesthesia using 2% lidocaine at the treatment sites. Cotton rolls were placed to isolate the target teeth.

#### 2.3.2. Endodontic Infection Protocol

Access cavities were prepared on the selected teeth using a #330 carbide bur. Pulp tissue was extirpated using a barbed broach. The working length (WL) for each root canal was determined using a #10 K-file in conjunction with an electronic apex locator (Root ZX, Morita, Kyoto, Japan), and was subsequently confirmed radiographically with a #15 H-file; the #15 H-file was then designated as the initial apical file (IAF). The canals were irrigated with sterile saline, then dried with sterile paper points.

To induce infection, 100 µL of *E. faecalis* suspension (10^6^ CFU/mL) was inoculated into each canal using a 30-gauge needle attached to a sterile syringe. To promote apical penetration of the bacteria, a size #8 hand K-file was inserted 1 mm beyond the WL. The access cavity was sealed with Caviton (GC Corporation, Tokyo, Japan) and subsequently restored with Single Bond Universal and Filtek™ Z350 XT composite resin (3M, St. Paul, MN, USA) in accordance with the manufacturers’ instructions. Clinical photographs and radiographs were taken to confirm completion.

#### 2.3.3. Experimental Treatment

**Figure 2:**
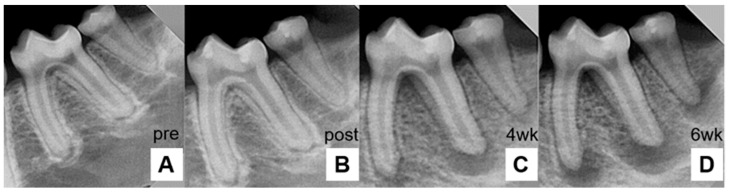
Time-dependent radiographic changes in the periapical lesion. (**A**) Pre-experiment, (**B**) post-bacterial inoculation, (**C**) 4 weeks after inoculation, and (**D**) 6 weeks after inoculation. Bacteria were injected into a healthy periapical environment. After 4 weeks, a distinct periapical lesion appeared. After 6 weeks, the lesion had enlarged markedly.

At 6 weeks post-inoculation, the development of chronic periapical lesions was confirmed by the presence of radiolucent areas around the apex (Figure 2).

The master apical file (MAF) size was determined to be three sizes larger than the initial apical file (IAF). For canals with a working length of 25 mm or less, canal shaping was performed using the ProTaper Gold system (Dentsply Sirona, Tulsa, OK, USA) in the sequence of Sx, S1, S2, F1, F2, F3, F4, and F5. For canals longer than 25 mm, coronal flaring was first performed with the Sx file, followed by apical preparation using stainless steel K-files with a step-back technique. At the final stage of instrumentation, an apically fitted H-file was used to refine the apical preparation and remove residual dentinal wall irregularities. Instrumentation up to size F5 was deemed sufficient under the present experimental conditions because the apical region had been enlarged sufficiently beyond the initial apical size, and clean dentin debris on the file flutes was confirmed at the final stage of instrumentation, indicating contact with sound dentinal walls.

Root canal irrigation was performed with 2.5% sodium hypochlorite (NaOCl) during instrumentation. The canals were subsequently soaked in NaOCl for 10 min and subjected to ultrasonic activation using an EndoActivator (Dentsply Sirona, Charlotte, NC, USA) to enhance apical disinfection. The canals were then irrigated with Hexamedine, followed by a second ultrasonic activation cycle. A final saline rinse was performed to remove any residual irrigant. Irrigation solutions were delivered using a syringe fitted with a 27-gauge needle to ensure penetration into the canal space. The canals were then dried with paper points and obturated with the allocated material (Vitapex^®^, Calcipex, or NaI paste), as illustrated in Figure 1. Vitapex^®^ and Calcipex were delivered using manufacturer-supplied syringes, whereas the experimental NaI paste was injected using a syringe fitted with a 27-gauge needle. After intracanal medicament placement, the access cavities were sealed with Caviton and restored with composite resin. Postoperative intraoral photographs and periapical radiographs were taken to evaluate the quality of the root canal filling.

Follow-up radiographs were taken at 4, 8, 12, and 16 weeks after obturation to monitor healing and root resorption. Periapical radiographic images were obtained using a portable X-ray unit (Model REMEX-T100, REMEDI, Seoul, Republic of Korea) at an exposure setting of 70 kV, 2 mA, 0.05 s. The images were processed and reviewed using REMEDIC/R-VIEWER software (Version 1.0, REMEDI, Seoul, Republic of Korea).

#### 2.3.4. Euthanasia

No adverse events or notable complications were observed in the experimental subject throughout the study period. At 16 weeks post-obturation, general anesthesia was re-induced with an intramuscular injection of a 1:1 mixture of Zoletil 50 and Rompun at 0.2 mL/kg. After confirming deep general anesthesia, euthanasia was completed by rapid intravenous administration of potassium chloride injection (KCl 150 g/mL, JW Pharmaceutical Co., Seoul, Republic of Korea) at 1 mL/10 kg, in line with the current guidelines. Death was confirmed by the absence of heartbeat and respiration and loss of the corneal reflex.

#### 2.3.5. Histological Processing and Staining

Paraffin-embedded tissue blocks were sectioned at a thickness of 3–4 μm using a microtome (HM-325, Thermo Scientific, Shanghai, China). The sections were mounted onto poly-L-lysine-coated glass slides (P0425, Sigma-Aldrich, St. Louis, MO, USA) and dried prior to staining. For deparaffinization, the tissue sections were immersed in xylene (Daejung Chemicals, Chungcheongbuk-do, Republic of Korea) and subsequently rehydrated through a graded ethanol series of 100%, 95%, and 70% ethanol (Daejung Chemicals, Jeollabuk-do, Republic of Korea).

##### Decalcification and Histological Section Preparation

After fixation, tissue specimens were transferred to fresh 10% neutral buffered formalin (Duksan Pure Chemicals, Gyeonggi-do, Republic of Korea) and fixed for an additional 2–3 days. Subsequently, the specimens were decalcified in a 10% ethylenediaminetetraacetic acid (EDTA) solution (pH 7.0). The decalcification solution was replaced at 1-week intervals, and the progress of decalcification was monitored by assessing calcium removal from bone and dental tissues.

Upon the completion of decalcification, the specimens were thoroughly rinsed under running tap water for 6 h. The tissues were then dehydrated through a graded ethanol series (70%, 80%, 90%, and 100%; Daejung Chemicals, Jeonju, Republic of Korea). Following dehydration, tissue infiltration was performed using xylene (Daejung Chemicals, Chungcheongbuk-do, Republic of Korea) as an intermediate clearing agent, after which the specimens were infiltrated with molten paraffin wax (Cat. No. 327204, Sigma-Aldrich, St. Louis, MO, USA).

The infiltrated tissues were embedded in paraffin using embedding molds, with the region of interest oriented downward to ensure optimal sectioning. Paraffin blocks were then sectioned at a thickness of 3–4 μm using a microtome (HM-325, Thermo Scientific, Shanghai, China). The sections were mounted onto poly-L-lysine-coated glass slides (P0425, Sigma-Aldrich) for subsequent histological staining and analysis.

##### Hematoxylin and Eosin (H&E) Staining

Paraffin sections prepared as described above were deparaffinized in xylene (Daejung Chemicals) and rehydrated through a graded series of 100%, 95%, and 70% ethanol (Daejung Chemicals). The sections were stained with Harris hematoxylin (BBC Biochemical, Mount Vernon, WA, USA) for 10 min, followed by rinsing under running tap water. Counterstaining was performed with eosin Y solution (Cat. No. 318906, Sigma-Aldrich, USA) for 2 min. The stained sections were dehydrated through graded ethanol solutions (70%, 95%, and 100%) and mounted with a mounting medium (SP15-500, Thermo Fisher Scientific, Waltham, MA, USA) under cover glass.

##### Masson’s Trichrome (MT) Staining

For MT staining, rehydrated sections were mordanted in Bouin’s solution (BBC Biochemical, Mount Vernon, WA, USA) at 53 °C for 60 min in a drying oven. The slides were allowed to cool at room temperature for 15 min and rinsed under running tap water for 10 min. Nuclear staining was performed using Harris hematoxylin (BBC Biochemical, USA) for 5 min, followed by rinsing with tap water. The sections were then stained with Biebrich scarlet-acid fuchsin solution (Trichrome staining kit, BBC Biochemical) for 12 min and rinsed with tap water. Differentiation was carried out using phosphotungstic-phosphomolybdic acid solution (Trichrome staining kit, BBC Biochemical) for 10 min, followed by staining with aniline blue solution (Trichrome staining kit, BBC Biochemical) for 3 min. Final differentiation was performed by immersing the sections in 1% acetic acid for 1 min. The sections were dehydrated through graded ethanol solutions (70%, 95%, and 100%) and mounted with a mounting medium (SP15-500, Thermo Fisher Scientific) under cover glass. In Masson’s trichrome-stained sections, collagen fibers were stained blue, muscle fibers and cytoplasm red, and nuclei black.

##### Immunohistochemistry

Paraffin-embedded tissue sections prepared as described above were mounted onto APS-coated glass slides (HMA-APS-11, Matsunami, Osaka, Japan), deparaffinized in xylene, and rehydrated through a graded ethanol series. For heat-induced epitope retrieval, sections were immersed in either sodium citrate buffer (pH 6.0) and heated in a microwave oven for 5 min, followed by incubation in the buffer for 30 min, cooling at room temperature for 10 min, and rinsing with 1× PBS. Endogenous peroxidase activity was blocked by incubation with 3% hydrogen peroxide for 10 min. To prevent nonspecific binding, sections were incubated with blocking buffer containing normal goat serum (Abcam, Cambridge, UK) for 1 h at room temperature. The sections were then incubated overnight at 4 °C with the following primary antibody: anti-myeloperoxidase (MPO), a marker of neutrophils (1:2000; A0398, Dako, Glostrup, Denmark). After washing with 1 × PBS, sections were incubated with horseradish peroxidase (HRP)-conjugated secondary antibodies (1:500; ab205719 or ab205718, Abcam) for 1 h at room temperature. Immunoreactivity was visualized using 3,3′-diaminobenzidine (DAB) substrate (1:500; DakoCytomation, Glostrup, Denmark, K3468) for 1 min, followed by counterstaining with Harris hematoxylin (BBC Biochemical). The stained sections were subsequently rinsed, dehydrated, and mounted for microscopic evaluation.

#### 2.3.6. Quantitative and Semiquantitative Histopathologic Evaluation

All stained slides were digitally scanned using a whole-slide imaging system (PANNORAMIC MIDI II; 3DHISTECH, Budapest, Hungary) for subsequent quantitative and semi-quantitative analyses.

##### Quantitative Assessment of Inflammatory Area and Perimeter

Quantitative analysis of the inflammatory lesion area and perimeter was performed on hematoxylin and eosin (H&E)-stained slides. Under the supervision of an experienced oral pathologist, regions exhibiting inflammatory changes were identified on whole-slide images. Digital slide evaluation was conducted using Slide Viewer software (Build 2.7.0.191696; 3DHISTECH, Budapest, Hungary). Using this platform, the perimeter and area of the inflammation-associated regions were manually delineated and quantitatively measured.

##### Semi-Quantitative Evaluation of Stromal Fibrosis

Stromal fibrosis in the periapical region was evaluated on Masson’s trichrome (MT)-stained sections using light microscopy (DP28; Olympus, Tokyo, Japan). Fibrosis was assessed using a semi-quantitative scoring system ranging from 0 to 3, where a score of 0 indicated no or minimal collagen deposition, 1 indicated mild fibrosis characterized by scattered thin collagen fibers, 2 indicated moderate fibrosis defined as collagen fibers occupying up to approximately 60% of the evaluated area, and 3 indicated severe fibrosis defined as dense collagen bundles occupying more than 60% of the evaluated area. For each specimen, five high-power regions of interest (ROIs) were selected within the periapical area, and a fibrosis score was assigned to each ROI. The mean fibrosis score derived from the five ROIs was calculated and used for subsequent statistical analysis.

##### Quantification of Inflammatory Cells

Inflammatory cell infiltration was quantified on H&E-stained or IHC sections. For each experimental group, two representative high-power ROIs were selected from the periapical inflammatory region. Within each ROI, inflammatory cells were manually counted and classified into polymorphonuclear neutrophils (PMNs), lymphocytes, plasma cells, and macrophages based on established morphological criteria. The mean value obtained from the two ROIs was calculated and used for statistical analysis.

#### 2.3.7. Statistical Analysis

Blinding was not possible during treatment because the operator performing the experimental procedures was required to place the assigned material. However, radiographic assessment, histologic evaluation, and statistical analysis were conducted using anonymized coded group labels (e.g., Group 1–5), and the actual treatment assignments were concealed until completion of the evaluations.

**Figure 3:**
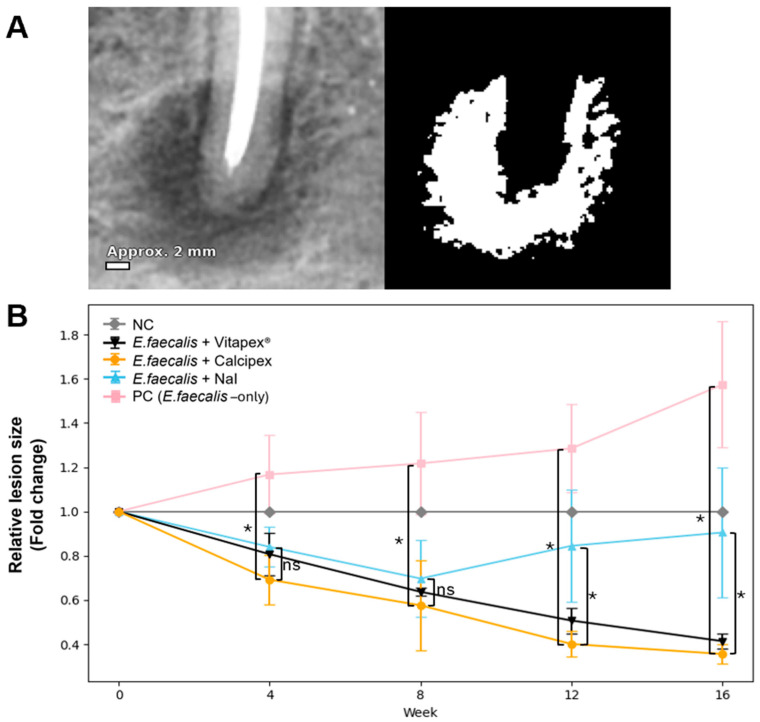
Radiographic assessment of periapical lesion size. (**A**) Quantitative analysis of defect size was performed using ImageJ software. To minimize measurement errors caused by variations in vertical and horizontal angulation during periapical radiography, images were calibrated prior to analysis. For each root canal, the lesion area was delineated, and its size was calculated based on pixel counts. An approximate scale reference is provided for visual guidance. (**B**) Changes in relative lesion size over time by group (mean ± SD). Baseline (week 0) was set to 1. The PC group showed a continuous increase in lesion size, whereas the Vitapex^®^ and Calcipex groups showed continuous decreases. The NaI paste group showed a decreasing trend until week 8, followed by a gradual increase thereafter. Intergroup differences were analyzed using one-way ANOVA followed by Tukey’s post hoc test. Vertical brackets on the left indicate comparisons between the PC group and each treatment group, while brackets on the right indicate comparisons between the NaI paste group and both the Vitapex^®^ and Calcipex groups. * *p* < 0.05; ns, not significant.

Radiographs were first calibrated in ImageJ (version 1.53; National Institutes of Health, Bethesda, MD, USA) to convert pixels to millimeters using a known reference. Canal-specific images were extracted for analysis. The periapical radiolucent area on each radiograph was delineated and quantified (mm^2^) using ImageJ by a single experienced blinded operator (Figure 3). Statistical analyses were performed with SPSS software (version 26.0; IBM Corp., Armonk, NY, USA). Intergroup differences at each time point (4, 8, 12, and 16 weeks) were evaluated using one-way analysis of variance (ANOVA), followed by Tukey’s post hoc test for multiple comparisons. Intragroup changes over time (0–16 weeks) were analyzed using repeated-measures ANOVA, with the Greenhouse–Geisser correction applied when the sphericity assumption was violated. When normality assumptions were not met, the Friedman test was used as a non-parametric alternative. Statistical significance was set at *p* < 0.05.

Because the study involved multiple root canals within a single animal, complete statistical independence could not be assumed. Therefore, each root canal was treated as an analytical unit for exploratory within-animal comparison, and no formal adjustment for within-animal clustering was applied.

## 3. Results

### 3.1. Radiographic Evaluation

#### 3.1.1. Sample Characteristics and Grouping

Initially, 36 teeth were enrolled in the study. After excluding teeth with restoration loss due to severe attrition that resulted in material leakage, as well as those with overlapping lesions that hindered accurate assessment, 31 teeth were ultimately analyzed. Among them, 12 were anterior teeth, 6 were single-rooted molars, and 13 were multi-rooted molars, comprising a total of 44 root canals. The distribution of root canals by group is presented in Table 2.

#### 3.1.2. Intergroup Comparison

For analysis, the lesion size at the time of medication application (week 0) was designated as the baseline (set to 1), and subsequent values were expressed as ratios relative to this baseline. The mean ± standard deviation values and statistical comparisons are presented in Figure 3.

At 4 and 8 weeks, lesion size increased in the positive control (PC) group compared with the baseline, whereas the Vitapex^®^, Calcipex, and NaI paste groups showed reductions. However, no statistically significant differences were observed among the experimental groups at these time points (*p* = 0.318 at 4 weeks; *p* = 0.287 at 8 weeks).

At 12 weeks, lesion size in PC continued to increase, while Vitapex^®^ and Calcipex showed further reductions. The NaI paste group showed a slight overall reduction compared with the baseline, although enlargement was observed in some canals. One-way ANOVA revealed significant intergroup differences (*p* < 0.001).

At 16 weeks, lesion size remained elevated in the PC group, whereas the Vitapex^®^ and Calcipex groups maintained reduced lesion sizes. In the NaI paste group, the mean lesion size remained close to the baseline, with enlargement observed in some canals. Significant intergroup differences were confirmed by one-way ANOVA followed by Tukey’s post hoc test (*p* < 0.001).

#### 3.1.3. Intragroup Comparison

**Figure 4:**
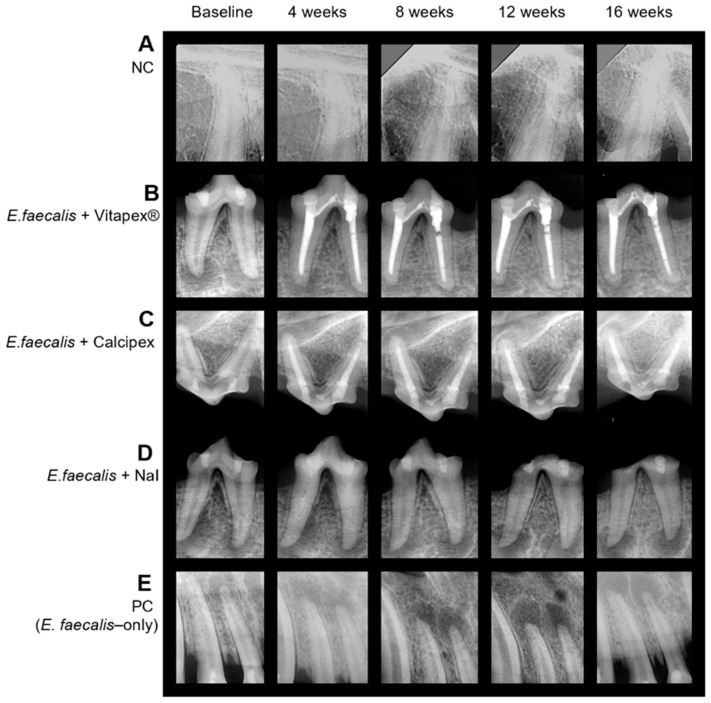
Representative radiographic changes in periapical lesions. (**A**) NC group. No radiographic changes were observed throughout the experimental period. (**B**) Vitapex group. Lesion size and radiolucency gradually decreased over time. (**C**) Calcipex group. Lesion size and radiolucency gradually decreased over time. (**D**) NaI paste group. After obturation with NaI paste, lesion size and radiolucency initially decreased but increased again at 12 and 16 weeks. The radiopacity of the intracanal NaI paste, which was clearly visible at 4 weeks, decreased from 8 weeks onward. Severe coronal wear was observed after 12 weeks. (**E**) PC group. Lesion size and radiolucency gradually increased over time.

Within-group changes in lesion size over time were statistically significant (*p* < 0.05), except in the negative control (NC) group (Figure 4). The results of the post hoc comparisons were as follows:

NC: Lesion size remained stable throughout the experimental period, with no statistically significant changes observed at any time point (*p* > 0.05).
Vitapex^®^: Significant reductions were observed at all time points compared with the baseline, with clear differences between successive intervals (*p* < 0.001).
Calcipex: Similar to the Vitapex^®^ group, significant reductions from the baseline were observed at all time points, and additional differences were detected between intermediate time points (*p* < 0.001).
NaI paste: A significant decrease from the baseline was observed at 4 and 8 weeks, followed by significant increases at the subsequent time points (*p* < 0.001).
PC: A significant increase was observed at all time points compared with the baseline, and significant differences were also noted between intermediate time points (*p* < 0.001).

### 3.2. Histological Analysis

#### 3.2.1. Quantitative Analysis of Inflammatory Area and Perimeter

**Figure 5:**
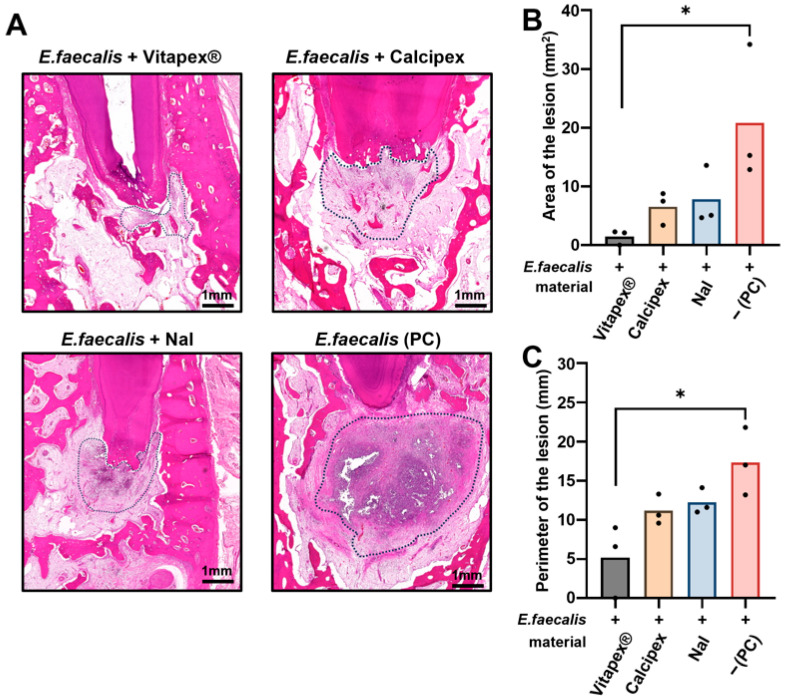
Quantitative evaluation of periapical inflammatory area and perimeter at 16 weeks according to the root canal filling material used. (**A**) Representative hematoxylin and eosin (H&E)–stained images showing the periapical inflammatory area according to the type of root canal filling material; the encircled area indicates the inflammatory region. (**B**) Quantitative analysis of inflammatory area, showing a significantly smaller lesion area in the Vitapex® group than in the PC group (* one-way ANOVA with Tukey’s multiple comparisons, *p* < 0.05). (**C**) Quantitative analysis of inflammatory perimeter, showing a significantly smaller lesion perimeter in the Vitapex® group than in the PC group (* one-way ANOVA with Tukey’s multiple comparisons, *p* < 0.05).

Quantitative analysis of inflammatory lesion area and perimeter was performed on H&E-stained sections. Although the perimeter has limitations due to its greater variability, it was included because it may provide additional descriptive information regarding lesion contour in conjunction with the lesion area. All experimental groups exhibited an increasing trend in inflammatory area and perimeter in the order of Vitapex^®^, Calcipex, NaI, and PC. One-way analysis of variance (ANOVA) with multiple comparisons revealed a statistically significant difference in both inflammatory area and perimeter between the Vitapex^®^ group and the PC group (*p* < 0.05) (Figure 5). No statistically significant differences were observed among the other intergroup comparisons.

#### 3.2.2. Semi-Quantitative Evaluation of Stromal Fibrosis

**Figure 6:**
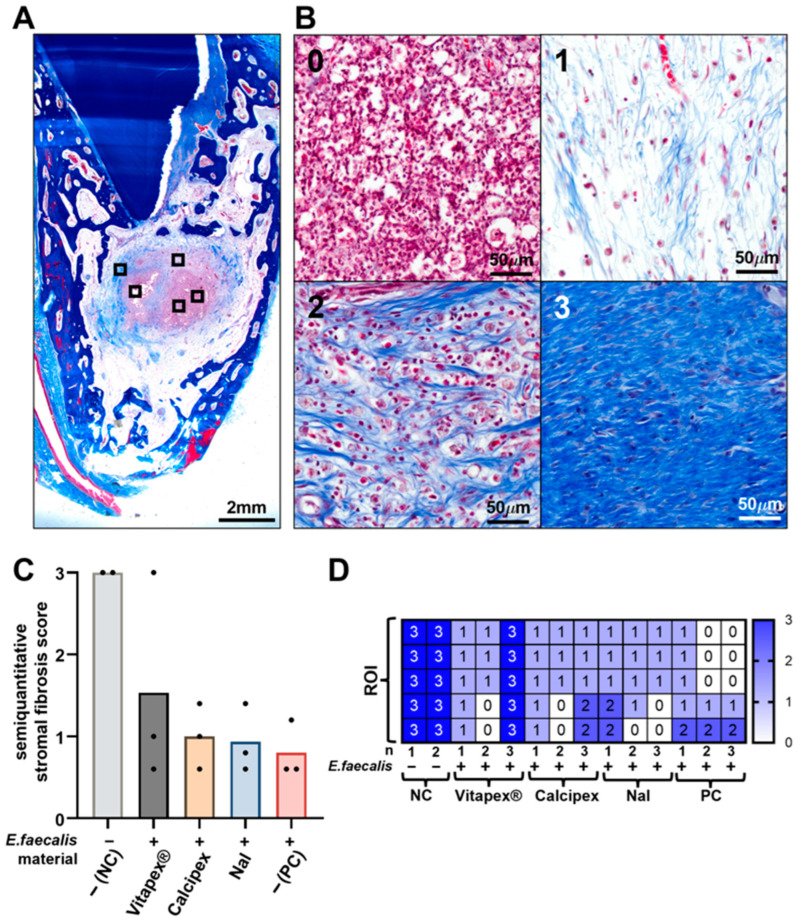
Semi-quantitative evaluation of stromal fibrosis at 16 weeks in the periapical region using MT staining. (**A**) Schematic representation of the method used to assess stromal fibrosis in the periapical inflammatory region according to the root canal filling material used. Five ROIs were selected within the periapical area (boxed area, ROIs), and a stromal fibrosis score was assigned to each ROI based on MT staining. The mean value of the five ROIs was calculated for each specimen. (**B**) Representative MT-stained histologic images illustrating stromal fibrosis according to the semi-quantitative scoring system (numbers, stromal fibrosis scores). (**C**) Semi-quantitative stromal fibrosis scores according to the root canal filling material used. A decreasing trend in stromal fibrosis was observed in the order of Vitapex^®^, Calcipex, NaI, and PC; however, no statistically significant differences were detected among the groups. (**D**) Distribution of stromal fibrosis scores across individual ROIs for each experimental group (one-way ANOVA with Tukey’s multiple comparisons, *p* < 0.05).

Stromal fibrosis was assessed using MT staining and a semi-quantitative scoring system. Fibrosis scores showed a decreasing trend in the order of Vitapex^®^, Calcipex, NaI, and PC. Despite this trend, no statistically significant differences in fibrosis scores were detected among the experimental groups (Figure 6C).

### 3.3. Quantification of Total Inflammatory Cell Infiltration

**Figure 7:**
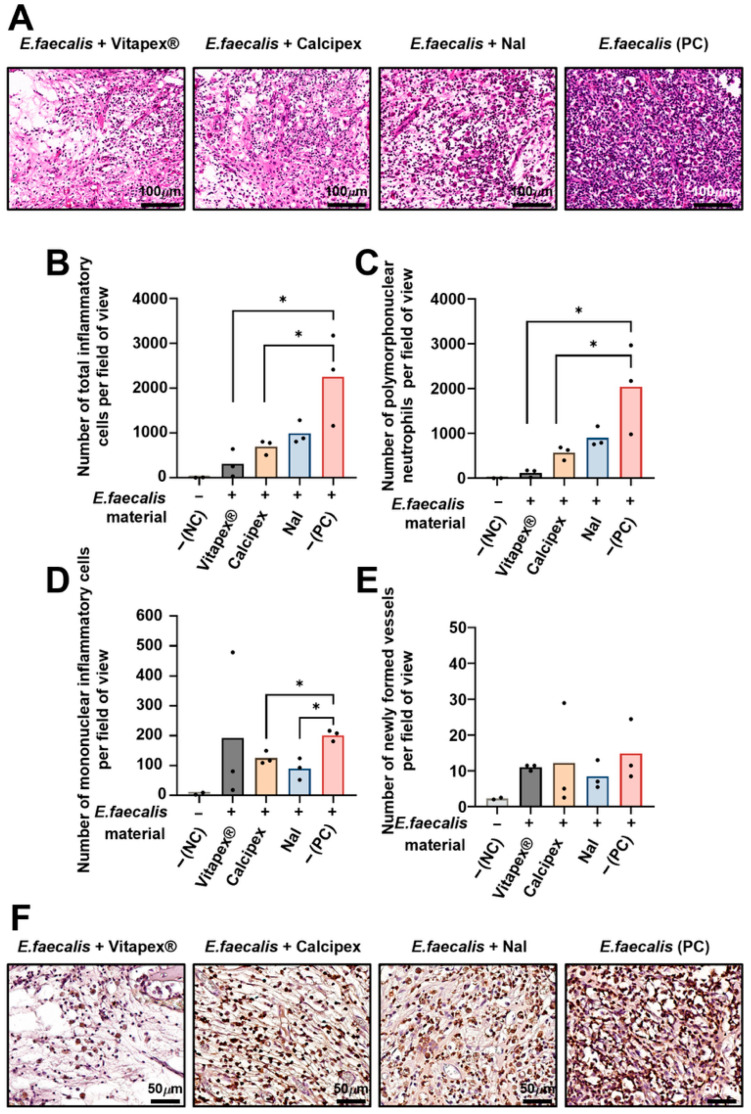
Quantitative analysis of inflammatory cell populations and neovascularization at 16 weeks in the periapical region. Quantitative assessments were performed on H&E-stained sections. (**A**) Representative H&E-stained histologic images. (**B**) Total inflammatory cell counts, demonstrating an increasing trend from the Vitapex^®^ and Calcipex groups to the NaI and PC groups, with statistically significant differences observed between the Vitapex^®^ and PC groups and between the Calcipex and PC groups (* one-way ANOVA followed by Tukey’s multiple comparisons test, *p* < 0.05). (**C**) polymorphonuclear neutrophil (PMN) counts, demonstrating an increasing trend from the Vitapex^®^ and Calcipex groups to the NaI and PC groups, with statistically significant differences observed between the Vitapex^®^ and PC groups and between the Calcipex and PC groups (* one-way ANOVA followed by Tukey’s multiple comparisons test, *p* < 0.05). (**D**) mononuclear inflammatory cell counts (lymphocytes, plasma cells, and macrophages), demonstrating significantly reduced in the Calcipex and NaI groups compared with the PC group (* Student’s *t*-test, *p* < 0.05). (**E**) the number of blood vessels in the periapical region showing no statistically significant differences in neovascularization were detected among the experimental groups (one-way ANOVA with multiple comparisons). Total inflammatory cell and PMN counts exhibited (one-way ANOVA followed by Tukey’s multiple comparisons test, *p* < 0.05). (**F**) Representative immunohistochemical staining for myeloperoxidase (MPO), a neutrophil marker, demonstrating MPO-positive inflammatory cells consistent with the quantitative findings indicating neutrophil predominance.

Total inflammatory cell counts showed an increasing trend in the order of Vitapex^®^, Calcipex, NaI, and the PC group. One-way analysis of variance with multiple comparisons revealed statistically significant differences between the Vitapex^®^ and PC groups, as well as between the Calcipex and PC (*p* < 0.05) (Figure 7B). Similarly, PMN counts, indicative of acute inflammatory responses, increased sequentially in the order of Vitapex^®^, Calcipex, NaI, and PC. Statistically significant differences were observed between the Vitapex^®^ and PC groups and between the Calcipex and PC groups (one-way ANOVA with multiple comparisons, *p* < 0.05) (Figure 7C). In contrast, mononuclear inflammatory cell infiltration, representing chronic inflammatory responses, was reduced in the Calcipex and NaI groups compared with the PC group, with these differences reaching statistical significance as determined by Student’s *t*-test (*p* < 0.05) (Figure 7D). The number of newly formed blood vessels did not differ significantly among any of the experimental groups (Figure 7E). Quantitative analysis of inflammatory cells revealed that the majority of infiltrating inflammatory cells were neutrophils. Accordingly, immunohistochemical staining for MPO, a representative neutrophil marker, was performed, and MPO-positive cells showed a distribution pattern consistent with the quantitative findings (Figure 7F).

## 4. Discussion

The present study demonstrated that Vitapex^®^ showed continuous radiographic healing throughout the 16-week observation period. This observation is consistent with reports that iodoform-containing calcium hydroxide paste can be associated with mineralized barrier formation during apical healing [22]. These favorable short-term outcomes align with previous clinical findings demonstrating effective periapical repair and predictable resorption behavior during physiological root resorption. However, accumulating evidence suggests that the long-term success of iodoform-based materials may be limited compared with that of alternative materials. A recent systematic review reported that although clinical success rates are comparable within the first year, outcomes beyond 18 months tend to favor other materials such as zinc oxide-eugenol [23]. Taken together, these findings indicate that although iodoform-based pastes remain clinically valuable, improvements in long-term dimensional stability and sustained antimicrobial effectiveness are desirable, particularly in cases involving persistent bacterial contamination or extended healing periods. As the present findings were derived from a single-animal exploratory model, they should be interpreted as preliminary rather than definitive evidence.

In addition, histological analysis was performed in parallel with radiographic analysis in the present study. At 16 weeks, histological analysis revealed that the periapical inflammatory area and perimeter increased in the order of Vitapex^®^, Calcipex, and NaI, whereas the radiographic defect area showed a different trend, increasing in the order of Calcipex, Vitapex^®^, and NaI. While radiographic evaluation reflects the three-dimensional extent of periapical bone destruction, histologic analysis is inherently limited to two-dimensional sectional evaluation. Consequently, histopathologic measurements may not consistently capture the maximal lesion extent, particularly given the technical variability introduced during tissue processing, embedding, and sectioning. These methodological differences likely contribute to divergent interpretations of the radiographic and histologic findings and underscore the importance of complementary multimodal assessment when evaluating endodontic treatment outcomes.

Calcipex was selected as a comparator alongside Vitapex^®^ because it represents a widely used, non-iodoform, calcium hydroxide-based intracanal medicament [24]. Calcium hydroxide formulations offer an established safety profile, high alkaline pH, and diffusible hydroxyl-ion release, providing a clinically relevant comparator for evaluating the early antimicrobial and sealing performance of new materials. In the present study, the Calcipex arm demonstrated sustained antimicrobial activity over the observation period, supporting its use as a practical reference comparator.

Recent next-generation sequencing (NGS) studies have shown that treatment failure is typically polymicrobial rather than caused by a single species [25]. In addition to *E. faecalis*, taxa such as *Fusobacterium*, *Olsenella*, *Dialister*, *Veillonella,* and *Synergistetes* have been identified with high prevalence in refractory cases, highlighting the complexity of endodontic biofilms and the need for materials capable of providing sustained antimicrobial activity against diverse microbial communities [26]. Even so, *E. faecalis* remains among the most consistently recovered species in persistent and retreatment cases, making a standardized *E. faecalis* challenge a pragmatic reference standard for the early assessment of intracanal medicaments. We therefore adopted this single-species model to assess the antimicrobial action and sealing performance under controlled, reproducible conditions, while acknowledging that subsequent validation in multispecies biofilm systems and clinical settings is required to confirm generalizability.

In this study, teeth treated with an NaI-based root canal filling material initially showed a gradual reduction in lesion size until approximately 8 weeks post-treatment; however, re-enlargement of the lesions was observed during longer-term follow-up. In contrast, teeth treated with Vitapex^®^ and Calcipex under the same conditions demonstrated a continuous reduction in lesion size. This difference may be attributable to the composition and mechanisms of action of the intracanal medicaments. These findings suggest that although NaI formulations showed a favorable early radiographic healing response, this trend may reflect transient antimicrobial or anti-inflammatory potential, as supported by previous studies. However, because these effects were not directly quantified in this study, these explanations should be interpreted as preliminary working hypotheses rather than definitive mechanistic conclusions.

Technical limitations during the filling procedures should be considered when interpreting the NaI-related findings. Unlike commercially available medicaments with dedicated syringes and tips, the experimental NaI paste lacked a controlled delivery system, which may have interfered with uniform placement. Adequate and dense filling to the apical region is critical for antimicrobial efficacy and clinical success [27]. Without proper delivery devices, issues such as apical extrusion or void formation may occur [28], which may in turn compromise sealing quality and contribute to the persistence or regrowth of residual infection. To fully assess the potential of NaI, more uniform delivery using dedicated dispensers [29], paste carriers, or Lentulo spirals [30] should be considered in future studies.

Sodium iodide (NaI), an iodine-based compound, can contribute to the suppression of intracanal pathogens through its antimicrobial properties. Choi et al. [10] reported that high-concentration NaI formulations (D30) significantly inhibited osteoclast formation and maintained lower levels of pro-inflammatory gene expression compared with lower concentrations. Such effects may have contributed to the early modulation of inflammation and reduction in lesion size. However, NaI exerts its antimicrobial action primarily through oxidative mechanisms, and the effective concentration may decline over time, permitting the regrowth of residual bacteria or recovery of biofilms. Notably, *E. faecalis*—a facultative anaerobe capable of surviving under unfavorable conditions—exhibits resistance via biofilm formation and other defense mechanisms [31], which may explain the recurrence of periapical lesions observed in the present study.

The physicochemical properties of NaI may also have played a role. As a water-soluble salt, NaI exhibits both solubility and hygroscopicity. An optimal degree of solubility is desirable for primary root canal filling materials to accommodate physiological root resorption. However, prolonged exposure to oral fluids may lead to degradation or dissolution, thereby diminishing antimicrobial efficacy and increasing susceptibility to reinfection [32]. In the present study, a lanolin-based optimized NaI formulation was used to reduce the excessive solubility previously reported in earlier preparations. Park et al. [12] demonstrated a relatively low solubility of approximately 2.69%, which meets the requirements of ISO 6876 for root canal sealing materials [21] but remains higher than that of Vitapex^®^ (0.34). This difference may hypothetically have influenced the outcomes. Radiographically, the gradual loss of radiopacity is hypothesized to result from iodine dissolution and leaching over time. Because iodine contributes to radiopacity by virtue of its high atomic number [33], its leaching may not only reduce radiographic contrast [13], but also compromise long-term antimicrobial activity. These interpretations remain hypothetical, because material dissolution, iodine release or leaching, and residual intracanal bacterial burden were not directly measured in the present study.

Tissue responses should also be taken into consideration. Choi et al. [10] reported that NaI (D30) suppressed osteoclastogenesis and reduced the expression of inflammatory mediators, but cytotoxicity was observed at 100% extract concentrations. Although the lanolin-based formulation used in this study was designed to minimize such cytotoxicity, in vivo factors such as drug concentration, leakage, and subclinical infection may still have contributed to chronic low-grade inflammation or delayed healing. Additionally, biomechanical factors such as occlusal forces, enzymatic degradation, and persistent stimuli could influence long-term tissue responses to NaI-based materials. For instance, Lee et al. [13] observed no inflammatory changes at 4 weeks in a canine model, but the limited observation period and differences from clinical conditions must be considered. In the present study, generalized attrition and occasional fractures of teeth or restorations were also observed, suggesting that excessive occlusal load and trauma may have contributed to the re-enlargement of lesions.

The biological characteristics and persistence-related resistance of *E. faecalis* also remain critical factors. Previous studies have consistently reported its high prevalence in retreatment and chronic periapical lesions, as well as its ability to invade dentinal tubules, form biofilms, and withstand nutrient deprivation and exposure to alkalis or oxidizing agents [34,35]. Stuart et al. [36] highlighted its persistence and biofilm-mediated resistance as major contributors to treatment failure, a finding corroborated by numerous systematic reviews. More recent studies suggest that conventional irrigants and filling strategies are insufficient to eradicate *E. faecalis* biofilms, highlighting the need for novel antimicrobial approaches [37]. Although NaOCl and chlorhexidine were sequentially applied as irrigants in this study [38], and ultrasonic activation (EndoActivator) was used to enhance canal debridement [39], re-enlargement of lesions over time suggests that NaI’s limited persistence, inadequate biofilm penetration, or incomplete filling may have contributed to the outcomes. These observations suggest that in *E. faecalis*-associated infections, not only the initial antimicrobial efficacy but also biofilm penetration and long-term antimicrobial stability may be important for treatment success.

This study has several limitations. First, the present work was intentionally designed as an exploratory within-animal feasibility assessment, using multiple root canals within a single animal for comparative evaluation while minimizing animal use in accordance with the 3Rs principle of reduction [40,41,42,43]. Nevertheless, because only one animal was included, inter-individual biological variability could not be evaluated and statistical generalizability is limited. In addition, anatomical and biomechanical differences between the maxilla and mandible and host-related factors (e.g., bone characteristics and age) may have influenced the outcomes; therefore, independence across root canals should be interpreted cautiously. In addition, because no formal statistical adjustment for within-animal clustering was applied, the resulting significance estimates should be interpreted with caution. Second, group allocation was not randomized. Teeth were distributed among groups to achieve a balanced representation of tooth types (anterior, premolar, and molar), but the absence of randomization may have introduced potential allocation bias associated with anatomical variations. Third, radiographic evaluation was performed using two-dimensional images, which cannot fully represent the three-dimensional extent of periapical lesions. Although radiography is useful for longitudinal monitoring, it provides only indirect information regarding periapical healing. The addition of volumetric analysis using micro-CT in future studies would improve the accuracy and biological interpretation of the results. Fourth, the infection model employed a single bacterial species (*Enterococcus faecalis*), whereas clinical endodontic infections are polymicrobial. Although a bacterium-induced periapical lesion model enhances clinical relevance, it does not fully replicate the complex microbial diversity and host immune responses observed in humans. Fifth, the limited observation period precluded the evaluation of the long-term effects of NaI. Physiological root resorption in primary teeth is a dynamic process extending over months to years, and the compatibility of NaI with this process remains to be elucidated. Moreover, as this study was conducted on permanent teeth, further investigation is required to evaluate the tissue responses and resorption processes in primary teeth with developing permanent successors.

While previous in vitro and short-term in vivo studies have demonstrated favorable biological properties of optimized NaI formulations, clinical conditions encompass far greater variability. Systemic health, local immune responses, apical sealing, and microleakage all substantially influence healing outcomes. Even biologically favorable materials cannot ensure success if intracanal infection is not fully eradicated or reinfection occurs, leading to lesion recurrence.

These considerations highlight the need for comprehensive long-term research to validate and expand upon the present findings. Future investigations should employ larger sample sizes, randomized group allocation, multimicrobial inoculation models, and three-dimensional quantitative imaging. Furthermore, studies focusing on optimizing NaI concentration, solubility, release kinetics, and biological responses will be essential to achieve stable and sustained lesion reduction. Long-term animal experiments and well-designed clinical trials will ultimately be required to confirm the stability, safety, and efficacy of NaI-based root canal filling materials.

## 5. Conclusions

In conclusion, this study suggests that NaI paste induced an initial reduction in lesion size but exhibited less durable long-term antimicrobial activity than conventional filling materials. Nonetheless, the significant initial antimicrobial activity of NaI remains promising. With improvements in formulation and the use of appropriate delivery techniques, NaI-based materials may be developed into clinically valuable alternatives for root canal filling. However, given the exploratory single-animal design, these findings should be interpreted as preliminary rather than definitive evidence.

## Figures and Tables

**Table 1:** Composition of the experimental materials.

Material	Composition
NaI paste (experimental)	Ca(OH)_2_ 28.75%, NaI 28.75%, Silicone oil 37.5%, Lanolin 5%
Vitapex^®^ (Neo Dental, Tokyo, Japan)	Iodoform 40.4%, Ca(OH)_2_ 30.3%, Silicone oil 22.4%, Others (inert) 6.9%
Calcipex II (Nishika, Shimonoseki, Japan)	Water-based Ca(OH)_2_ paste, BaSO_4_ (radiopacifier), purified water (vehicle); proportions not disclosed

**Table 2:** Experimental groups and sample size.

Group	Treatment Description	No. of Teeth (Anterior/Single-Rooted Molar/Multi-Rooted Molar)	Total Root Canals (n)
NC	No treatment	5 (2/0/3)	8
Vitapex^®^	*E. faecalis* inoculation + Vitapex^®^	7 (2/2/3)	10
Calcipex	*E. faecalis* inoculation + Calcipex	6 (3/1/2)	8
NaI	*E. faecalis* inoculation + NaI paste	7 (2/2/3)	10
PC	Inoculated with *E. faecalis* No medicament	6 (3/1/2)	8

Abbreviations: NC, negative control; PC, positive control.

## Data Availability

The original contributions presented in this study are included in the article/Supplementary Material. Further inquiries can be directed to the corresponding authors.

## Supplementary Materials

The following supporting information can be downloaded at: https://www.mdpi.com/article/10.3390/pharmaceutics18040493/s1, Figure S1: The PRIASE 2021 flowchart; Table S1: The PRIASE 2021 checklist; Table S2: The ARRIVE guidelines 2.0: author checklist; Table S3: Physicochemical properties of NaI-based L5 paste versus Vitapex^®^; Table S4: Lesion size over time by group.

## Abbreviations

The following abbreviations are used in this manuscript:

NaI	Sodium iodide
WL	Working length
IAF	Initial apical file
MAF	Master apical file
