# Supplementary material for: Exploratory Evaluation of a Sodium Iodide-Based Root Canal Filling Formulation in a Canine Model of Enterococcus faecalis-Induced Periapical Inflammation
Source: Pharmaceutics. 2026 Apr 17;18(4):493. doi: 10.3390/pharmaceutics18040493 (PMC13119582; doi:10.3390/pharmaceutics18040493)
Supplement: Supplementary file 1 [file pharmaceutics-18-00493-s001.zip › Supplementary Figure S1 PRIASE 2021 Final flowchart.pdf]

PRIASE 2021 Flowchart

AIM/HYPOTHESIS

Evaluated the feasibility of an NaI-based root canal filling material in a canine model of *Enterococcus faecalis*-induced periapical inflammation.

ETHICAL APPROVAL AND COMPLIANCE WITH ANIMAL WELFARE STANDARDS

Institutional Animal Care and Use Committee
(CRONEX-IACUC: 202410004)

AGE, GENDER, TYPE OF ANIMAL

12 months old, male, Mongrel dog

SAMPLES

A total of 36 teeth were included in the study: nine teeth from each quadrant

INTERVENTION / CONTROL GROUPS

Total N(root canal) = 44
NC - no treatment (n =8); Vitapex (n =10); Calcipex (n =8);
NaI paste (n =10); PC - *E. faecalis*-only (n =8)

OUTCOMES ASSESSED

Radiographic lesion size was assessed at 4, 8, 12, and 16 weeks post-obturation. Histological outcomes evaluated at 16 weeks included inflammatory area and perimeter, stromal fibrosis, inflammatory cell infiltration, and myeloperoxidase (MPO) expression.

METHODS AND WHO ASSESSES OUTCOMES

Radiographic lesion size was measured on serial radiographs obtained at 4, 8, 12, and 16 weeks post-obturation. Histological analysis at 16 weeks was performed to assess inflammatory area and perimeter, stromal fibrosis, inflammatory cell infiltration, and myeloperoxidase (MPO) expression. These outcomes were assessed by trained investigators using predefined criteria.

RESULTS WITH STATISTICAL ANALYSES

Radiographically, all treatment groups showed reduced lesion size relative to the pos-itive control. No significant differences were observed among the NaI, Vitapex®, and Calcipex groups at 4 and 8 weeks; however, significant differences emerged at 12 and 16 weeks ($p < 0.05$). The NaI group showed lesion reduction until week 8, followed by subsequent expansion thereafter, whereas the Vitapex® and Calcipex groups showed continuous lesion reduction over time. Histologically, the periapical inflammatory area increased in the order of Vitapex® < Calcipex < NaI < positive control ($p < 0.05$). MPO staining identified neutrophils as the primary inflammatory cells.

ADVERSE EVENTS

No systemic adverse events or unexpected distress were observed.

CONCLUSION(S)

NaI paste showed favorable early radiographic healing but limited long-term stability compared with conventional materials. With further optimization, it may have potential as an alternative root canal filling material. However, given the single-animal exploratory design, these findings should be interpreted as preliminary rather than definitive evidence.

FUNDING DETAILS

Supported by Dankook University (Dept. of Dentistry, Pediatric; University Innovation Support Program) and the National Research Foundation of Korea (RS-2021-NR06009; RS-2023-00220408)

CONFLICTS OF INTERSET

The authors declare that they have no conflict of interest.

***From:Nagendrababu V, Kishen A, Murray PE, Nekoofar MH, de Figueiredo JA, Priya E, Jayaraman J, PulikkotilSJ, Camilleri J, Silva RM, Dummer PM. PRIASE 2021 guidelines for reporting animal studies in Endodontology: a consensus-based development. Int Endod J. 2021 Jan 15. doi: 10.1111/iej.13477. https://onlinelibrary.wiley.com/doi/10.1111/iej.13477**

For further details visit: http://pride-endodonticguidelines.org/priase/
