# Supplementary material for: Exploratory Evaluation of a Sodium Iodide-Based Root Canal Filling Formulation in a Canine Model of Enterococcus faecalis-Induced Periapical Inflammation
Source: Pharmaceutics. 2026 Apr 17;18(4):493. doi: 10.3390/pharmaceutics18040493 (PMC13119582; doi:10.3390/pharmaceutics18040493)
Supplement: Supplementary file 1 [file pharmaceutics-18-00493-s001.zip › Supplementary Table S1 PRIASE2021checklist.pdf]

PRIASE 2021

Checklist of items to be included when reporting animal studies in Endodontology *

Section/ Topic	Item Number	Checklist Items	Reported on page number
Title	1a	The specific animal species and its health or disease status (sometimes called “animal model”) must be provided.	1
	1b	The specific test, field, subject and treatment of interest within the animal model must be provided.	1
Keywords	2a	Keywords such as “animal model” or “*in vivo* model” and the specific area(s) of interest must be provided.	1
Abstract	3a	The Introduction of the Abstract must explain the significance of the study.	1
	3b	The unambiguous aim(s) and objective(s) of the study must be provided.	1
	3c	The most important details of the animal and the experimental model must be provided.	1
	3d	Key details of the methodology must be provided.	1
	3e	The most relevant and important results must be presented succinctly including differences among the means, medians or modes of the dependent variables (treatment outcome and test results) and any significant P-values.	2
	3f	Succinct conclusions supported by the results must be provided.	2
Introduction	4a	The relevant background information must be provided using terminologies consistent with professional standards and previous publications.	2
	4b	The appropriateness of the selected animal model to address the aims and objectives of the study must be explained.	3
	4c	A justification of the reasons why the investigation was necessary using an animal model must be provided.	3
	4d	The unambiguous aim(s) and objectives(s) of the animal study must be provided.	3
Materials and Methods	5a	The reference number of the approval granted by the ethics board, such as an Institutional Review Board or Institutional Animal Care committee, must be provided along with a reference to the applicable institutional and/or national regulations that were enforced. Any identifying details about the authors institution should not be disclosed during the blind peer review.	~~3~~
	5b	The sample size must be justified by citing prior similar studies and/or be estimated by using statistical power calculations to ensure an adequate sample size is used to detect any significant differences and answer the research questions. This is to avoid making any type I and type II errors.	4
	5c	Details of how animal pain and disability was monitored and how animal suffering was prevented during all aspects of experimentation must be provided.	4

	5d	The job titles and qualifications of the animal caretakers must be provided.	4
	5e	Specific details of the animals must be provided, including their species, strain, immune system, breeding programme, age, weight, health status, and any special characteristics.	4
	5f	The experimental design must include details of the numbers of animals, numbers of experimental units (e.g. teeth), and timelines (e.g. 5, 30 and 60 days) used.	4
	5g	The primary outcome data measures or categories as well as any other secondary outcome data measures or categories that will be assessed must be provided.	12
	5h	Details must be provided on (1) steps in the interventions and treatments, (2) instruments, medicaments or device allocation, and (3) concealment and randomization prior to data collection.	4
	5i	Details regarding post-disease and post-operative care of the animals must be provided.	3-4
	5j	Details on the statistical analysis, statistical tests, type of software used, and steps taken to control, interpret success or failure, and to validate the accuracy of the data must be provided.	10
Results	6a	Average baseline characteristics of the animals (e.g. age, weight, gender, microbiological status) at the beginning of the experiment must be provided.	4
	6b	The results for each group of primary and secondary outcomes should describe the means, median or mode; as well as differences and their statistical significance.	11-17
	6c	All adverse events during the animal experimentation and the method of euthanasia must be reported.	7
	6d	Any changes made to the experimental protocols to prevent the occurrence of animal adverse health events, analgesic or other medication overdoses or underdoses, or unexpected deaths must be provided.	7
Discussion	7a	A discussion on how the methods and results are relevant to the study aims, and how the results support or dispute prevailing theories advocated in prior publications must be provided.	17-18
	7b	An objective presentation of the strengths and limitations of the animal model, study design, methods, materials, instruments, drugs and devices, and outcomes must be provided, including any biology/functional variability between the animal model and humans.	18-20
	7c	The potential influence of the results on future research plans must be discussed.	20
	7d	If appropriate, the impact the findings have on human health, treatments or healthcare must be explained.	20-21
Conclusion(s)	8a	A rational basis for the conclusion(s) must be provided, that is, they must be directly supported by the results of the study.	21
	8b	Explicit conclusion(s) from the study, including appropriate follow-up research ideas, must be provided.	21

Funding and support	9a	All funding, donations, assistance and support provided for the study must be reported.	21
Conflicts of interest	10a	An explicit statement on conflicts of interest must be provided.	21
Quality of images	11a	Details of the equipment (model, supplier, city, country), software (version, supplier city, country) and settings used to acquire image(s) must be described in the Methods and/or figure legend.	7
	11b	The reason why the image(s) was acquired and rationale for its inclusion in the manuscript must be provided in the text.	11,13-16
	11c	The circumstances (conditions) under which the image(s) was viewed and evaluated must be provided in the text.	9,10
	11d	The resolution, magnification and any important manipulation(s) on any image (e.g. brightness, image smoothing, staining etc.) must be described in the text or legend.	14-16
	11e	An interpretation of the findings (meaning and implications) from the image (s) must be provided in the text.	11,13-16
	11f	The legend associated with each image must clearly describe the subject matter specific feature(s) illustrated. Images of animals must describe their age and test duration, and other relevant features such as important anatomical landmarks and relevant features.	11,13-16
	11g	Arrow markers and relevant labels must be provided in image(s), if relevant, in order to identify key information.	14-16
	11h	The legend of each image must include an explanation whether it refers to pre-treatment, intra-treatment, post-treatment or post-sacrifice, and if relevant, how images were standardised over time.	11,13-16

***From: Nagendrababu V, Kishen A, Murray PE, Nekoofar MH, de Figueiredo JA, Priya E, Jayaraman J, Pulikkotil SJ, Camilleri J, Silva RM, Dummer PM. PRIASE 2021 guidelines for reporting animal studies in Endodontology: a consensus-based development. Int Endod J. 2021 Jan 15. doi: 10.1111/iej.13477. https://onlinelibrary.wiley.com/doi/10.1111/iej.13477.**

For further details visit: http://pride-endodonticguidelines.org/priase/
