# Supplementary material for: Exploratory Evaluation of a Sodium Iodide-Based Root Canal Filling Formulation in a Canine Model of Enterococcus faecalis-Induced Periapical Inflammation
Source: Pharmaceutics. 2026 Apr 17;18(4):493. doi: 10.3390/pharmaceutics18040493 (PMC13119582; doi:10.3390/pharmaceutics18040493)
Supplement: Supplementary file 1 [file pharmaceutics-18-00493-s001.zip › Supplementary Table S3.pdf]

Supplementary Table S3. Physiochemical properties of NaI-based L5 paste versus Vitapex.

Property	L5	Vitapex
Flow (mm)	17.27	17.97
Film thickness (mm)	0.132	0.032
Injection force (N)	45.26	505.72
Complex viscosity (mean, a.u.)	126.77	1170.72
Solubility (%)	1.15	0.34
pH	11.65	12.04

Values are mean (n = 3). Tests followed ISO 6876 (23 ± 2 °C)
Abbreviations: L5, lanolin-5% NaI-based paste; V, Vitapex; a.u., arbitrary units.
Information adapted from [11].
