# Supplementary material for: Exploratory Evaluation of a Sodium Iodide-Based Root Canal Filling Formulation in a Canine Model of Enterococcus faecalis-Induced Periapical Inflammation
Source: Pharmaceutics. 2026 Apr 17;18(4):493. doi: 10.3390/pharmaceutics18040493 (PMC13119582; doi:10.3390/pharmaceutics18040493)
Supplement: Supplementary file 1 [file pharmaceutics-18-00493-s001.zip › Supplementary Table S4.pdf]

Supplementary Table S4. Lesion size over time by group

Week	*E. faecalis*– only	NaI paste	Vitapex	Calcipex	*p* -value (ANOVA)
0	1	1	1	1	
4	1.167 ± 0.178 (1.025 –1.310)	0.84 ± 0.089 (0.784–0.895)	0.807 ± 0.097 (0.747 –0.868)	0.692 ± 0.112 (0.602 –0.781)	$p < 0.001$
8	1.217 ± 0.231 (1.032 –1.402)	0.697 ± 0.175 (0.588 –0.805)	0.637 ± 0.017 (0.627 –0.648)	0.576 ± 0.204 (0.413 –0.740)	$p < 0.001$
12	1.285 ± 0.200 (1.125 –1.445)	0.845 ± 0.253 (0.688 –1.002)	0.507 ± 0.058 (0.471 –0.543)	0.401 ± 0.058 (0.354 –0.448)	$p < 0.001$
16	1.574 ± 0.285 (1.346 –1.802)	0.905 ± 0.293 (0.724 –1.087)	0.414 ± 0.035 (0.392 –0.436)	0.356 ± 0.045 (0.320 –0.393)	$p < 0.001$

Values are mean ± SD (95% CI). p values calculated by one-way ANOVA at each time point.

$p < 0.05$ considered significant
